# Quality evaluation of medical care in clinical practice

**DOI:** 10.1590/1516-3180.2013.1313694

**Published:** 2013-06-01

**Authors:** Paulo Manuel Pêgo-Fernandes, Ricardo Mingarini Terra, Letícia Leone Lauricella, Benoit Jacques Bibas

**Affiliations:** I MD, PhD. Associate Professor, Discipline of Thoracic Surgery, Instituto do Coração (InCor), Hospital das Clínicas (HC), Faculdade de Medicina da Universidade de São Paulo (FMUSP), São Paulo, Brazil.; II MD, PhD. Collaborating Professor, Discipline of Thoracic Surgery, Discipline of Thoracic Surgery, Instituto do Coração (InCor), Hospital das Clínicas (HC), and Director of Thoracic Surgery Service, Cancer Institute of the State of São Paulo, Faculdade de Medicina da Universidade de São Paulo (FMUSP), São Paulo, Brazil.; III MD. Attending Physician, Thoracic Surgery Service, Cancer Institute of the State of São Paulo (FMUSP), São Paulo, Brazil.; IV MD. Postgraduate Student, Discipline of Thoracic Surgery, Instituto do Coração (InCor), Hospital das Clínicas (HC), Faculdade de Medicina da Universidade de São Paulo (FMUSP), São Paulo, Brazil.

Evaluation of the quality of medical care is an issue that is becoming topical in Brazil. However, this subject has been a recurrent issue in foreign medical associations. During the 1980s, the American medical community started to discuss the importance of comparative analysis of surgical results as a tool for improving the quality of surgical treatment. At that time, a need for a single register for combining data from different institutions arose, since the heterogeneity of the databases and definitions of outcomes in different services made this task impossible. However, the task of designing and implementing these registers was a challenge that required much leadership and funding.^1^^,^^2^^,^^3^

In 1986, action by the Health Care Financing Administration (HCFA), an agency of the American Medicare organization, indirectly propelled medical associations to implement such registers. In an attempt to increase the transparency of healthcare institutions, the HCFA published results relating to hospital mortality at different American institutions. Publication of these results was harshly criticized, because the HCFA’s data was based on administrative information without adjustments for the severity of the cases analyzed, which limited the comparability between the hospitals listed. Nevertheless, there were no alternative reliable sources of information and the program lasted until 1993, when it was discontinued because of problems relating to data analysis.^3^ This act of publication laid out the foundations of the issue of transparency in healthcare provision and also showed the need for standardized clinical data to be gathered for use in quality improvement.

Clinical registers are more precise than administrative registers with regard to generating data that, in the final analysis, will be used for improving quality and comparing performance between institutions. Administrative data often do not adequately identify the procedures of interest, do not include clinical variables that are critical for risk adjustment and often confound comorbidities and complications.^4^

Cardiac surgery was a pioneer in this regard and, in 1990, a database that would gather information on surgery for revascularization of the myocardium was started.^1^ This database analyzed a variety of outcomes and also gathered data that would enable risk adjustment. Over the years, many American institutions went on volunteering to participate and the data-gathering expanded to procedures other than revascularization surgery. Today, this database is an extremely important source of information for defining national standards for heart surgery, for improving quality in these various institutions and for the progress of science.^1^^,^^2^

The best example of the impact that registers of surgical outcomes have on the results and quality of surgical treatment comes from the experience of the Veterans’ Healthcare System (VHS) in the United States. In 1986, due to pressure from public opinion, with allegations of poor quality of medical care within this system, the American Congress determined that comparative results between VHS hospitals and other hospitals in the American private system should be published. Since there were no national databases, the VHS conducted the National Surgical Risk Study, which included 44 institutions linked to the VHS. Through this, outcomes for analysis and models for risk adjustment were established and validated.^5^

In 1994, the VHS started the National Surgical Quality Improvement Program, which gathers data on surgical patients and provides participating institutions with half-yearly bulletins containing the results, compared with national averages, thereby allowing administrators to provide better management. Since the Program began, there has been a 47% decrease in postoperative mortality and a 43% decrease in postoperative morbidity, thus placing the VHS in a prominent position within the American healthcare system. The American College of Surgeons, in association with the VHS, has expanded the Program to private institutions and similar results have been observed in this sector.^5^

Implementation of treatment registers and databases would bring benefits to society and to the Brazilian National Health System (Sistema Único de Saúde, SUS). Precise care quality criteria could direct healthcare policies towards areas in need. Moreover, it would be possible to determine reference centers for treatments on specific disorders, based on previously defined variants. As an example, surgical treatment for chronic pulmonary thromboembolic disease can be cited: for a center to be able to perform this procedure, it would be necessary to attain a minimum care quality level.

In addition to the care benefits, implementation of trustworthy databases with quality criteria would enable retrospective analysis on the current healthcare policies and on the impact of interventions on the evolution of the diseases studied. The clear example of this is the drastic reduction in surgical mortality that was evaluated by the American College of Surgeons, as cited earlier. In other words, real improvement in medical treatment is possible when clear and objective evidence-based outcomes are established for the therapy. Rigorous assessment of an institution and correct determination of the interventions necessary can only be undertaken in this manner.
